# Prognostic factors affecting clinical outcomes after arthroscopic rotator cuff repair: importance of functional recovery by 3 months after surgery

**DOI:** 10.1186/s13018-018-1014-8

**Published:** 2018-12-05

**Authors:** Yosuke Nakamura, Masafumi Gotoh, Yasuhiro Mitsui, Hidehiro Nakamura, Hiroki Ohzono, Takahiro Okawa, Naoto Shiba

**Affiliations:** Department of Orthopedic Surgery, Kurume University Medical Center, 155-1 Kokubu-machi, Kurume, Fukuoka 839-0863 Japan

**Keywords:** Rotator cuff tear, Arthroscopic rotator cuff repair, Prognostic factor, Functional recovery, Japanese Orthopedic Association score, Visual analog scale, Elevation

## Abstract

**Background:**

To examine important factors that affect clinical outcomes following arthroscopic rotator cuff repair (ARCR).

**Methods:**

Among 163 patients who underwent ARCR, we included 71 shoulders in 71 patients whose progress was monitored for > 2 years, postoperatively. We divided the patients into groups A (scores ≥ 83 points, 59 patients) and B (scores < 83 points, 12 patients) using the Japanese Orthopedic Association (JOA) score at 24 months. We then conducted univariate and multivariate analyses of pre- and postoperative (2 and 3 months, respectively) factors.

**Results:**

The mean JOA score for all patients significantly improved from 63.7 ± 11.5 points preoperatively to 90.3 ± 9.6 points at 24 months postoperatively (*P* < 0.05). However, there were no significant between-group differences in the preoperative scores. In addition, there were no significant differences in the postoperative re-tear rate. Univariate analysis revealed that the range of motion (preoperative abduction and postoperative elevation, abduction, internal rotation, and external rotation), muscle strength (external rotation 3 months postoperatively), postoperative pain level [visual analog scale (VAS) maximum score, 10 points], partial repair, Cofield classification, and preoperative width were significant factors (*P* < 0.05 for all factors). Multivariate and receiver operating characteristic curve analyses showed that VAS at 2 months postoperatively and elevation at 3 months postoperatively were significant factors.

**Conclusions:**

To obtain a JOA score of ≥ 83 points at 24 months postoperatively, following ARCR, a postoperative VAS of < 5 points at 2 months and postoperative elevation of ≥ 110° at 3 months should be achieved.

## Background

Rotator cuff tear requires a large amount of treatment [1, 2]. Arthroscopic rotator cuff repair (ARCR) is a well-developed, modern procedure that produces acceptable postoperative outcomes [3]. Cole et al. [4] showed that ARCR resulted in significant pain relief and improvements in active range of motion, strength, and function. In patients with ARCR, American Shoulder and Elbow Surgeons shoulder (ASES) scores, range of motion, and strength are improved regardless of postoperative tendon healing status [5]. In a recent systematic review, clinically significant improvements in patient-reported outcomes, range of motion, and strength were observed up to 1 year after ARCR [6].

Kurowicki et al. [7] showed that all patients who underwent ARCR demonstrated significant incremental improvements in pain, function, and motion at 3-, 6-, and 12-month time points, suggesting that the plateau of maximum recovery after ARCR occurred at 1 year with high satisfaction.

Several studies [8–10] reported preoperative prognostic factors associated with functional outcome after ARCR. Pécora et al. [10] showed that age is an independent predictive factor for obtaining good and excellent results after ARCR. Cho et al. [8] reported that the presence or absence of preoperative contractures affects internal and external rotation up to 6 months postoperatively, and contractures are also cited as prognostic factors. Ohzono et al. [9] reported that preoperative fatty degeneration of the subscapularis muscle and infraspinatus muscle affects clinical outcomes following the repair of large/extensive rotator cuff tears without re-tear.

In contrast, a few studies have focused on analyzing early postoperative prognostic factors associated with functional outcomes after ARCR. Kim et al. [11] demonstrated that stiffness of internal rotation at 3 months postoperatively affected the intensity pain pattern at the final follow-up. Tonotsuka et al. [12] reported that either an elevation less than 120° or external rotation less than 10° at 3 months postoperatively was associated with worse functional outcomes at 24 months postoperatively, suggesting the importance of functional results at the early phase of the postoperative course. In a recent systemic review [13], it was reported that postoperative re-tear tends to occur before 3 months postoperatively and significantly reduces both the University of California Los Angeles (UCLA) and ASES postoperative scores and strongly correlates with clinical outcomes. These reports consistently emphasized the importance of functional recovery at 3 months postoperatively and postoperative re-tear. Therefore, the purpose of the present study was to evaluate clinical results in patients with ARCR using the Japanese Orthopedic Association (JOA) score and to seek prognostic factors affecting clinical outcomes at 2 years postoperatively as evaluated by this score, extending to 3 months preoperatively to postoperatively.

## Methods

### Subjects

One hundred sixty-three patients underwent ARCR at our institution between January 2012 and December 2014. Inclusion criteria consisted of patients who (1) had ARCR and (2) were followed for 2 years postoperatively. Exclusion criteria consisted of patients who (1) had systemic disease and (2) had fractures around the shoulder. As a result, 71 patients with an average age of 63.4 ± 9.5 years were included in this study.

Patients underwent ARCR using a suture bridge technique under general anesthesia along with an interscalene block; subsequently, the appointed postoperative rehabilitation was performed. Functional evaluation was performed at 3, 6, 9, 12, and 24 months postoperatively. Evaluation by magnetic resonance imaging (MRI) was performed at 3, 12, and 24 months postoperatively. Depending on the patient’s JOA score at final follow-up, they were assigned to the satisfactory (group A: JOA score ≥ 83 points, *n* = 59) or unsatisfactory group (group B: the score < 83, *n* = 12). Univariate and multivariate analyses were used to analyze various clinical parameters.

### Surgical technique and postoperative regimen

Patients underwent ARCR in the beach chair position under general anesthesia along with an interscalene block. The torn cuff was repaired using the single-row, double-row, or suture bridge technique, depending on tendon mobility and tear configuration. For single-row repairs, one row of anchors was placed on the lateral aspect of the footprint, and the torn cuff was fixed with an interrupted suture. For double-row suture bridge repair, one row of anchors was placed on the medial aspect of the footprint with or without tying, and the torn cuff was transosseously fixed with a knotless anchor on the lateral aspect of the footprint. If needed, additional procedures, including capsular release, tenotomy or tenodesis of the long head of the biceps tendon, and distal clavicle excision, were performed. Moreover, acromioplasty was performed in all cases.

Patients were immobilized using a sling with an abduction pillow postoperatively, with the shoulder internally rotated at 30–40° and abducted at 20°. Passive range of motion (ROM) exercises of the shoulder commenced at postoperative day 4, and active ROM exercises were allowed at postoperative week 6. Isotonic muscle strengthening exercises were allowed at postoperative week 12.

### Functional assessment

JOA scores were used as clinical outcome measures. ROM was assessed using a goniometer, and muscle strength was measured using a hand-held dynamometer (Micro FET2; Hoggan Health Industry, West Jordan, UT, USA). Visual analog scale (VAS) scores were reported based on the patients’ subjective assessment. These measures were evaluated pre- and postoperatively.

### Structural assessment

Tear length, tear width, fatty degeneration, muscle atrophy preoperatively, and structural integrity postoperatively were examined using MRI, according to a previous report [14]. Postoperative “intact tendon” was defined as types I–III in the Sugaya classification [15]. The tear length and width were evaluated as the coronal and sagittal oblique distance on T2-weighted images, respectively [16]. The fatty degeneration of the rotator cuff muscles at the Y view were evaluated according to the Goutallier classification [17].

### Assignment of the satisfactory and unsatisfactory groups

Patients were divided into two groups according to the JOA score at final follow-up: the satisfactory group, composed of patients classified into the “good or excellent” criterion (≥ 83 points, *n* = 59), and the unsatisfactory group, composed of patients classified into the “poor or fair” criterion (< 83 points, *n* = 12) [18]. Various variables were used to analyze the association with satisfactory or unsatisfactory outcomes using univariate and multivariate analyses: patient’s age, sex, symptom duration, comorbidities, hand dominance, traumatic onset, worker’s compensation status, repair techniques, tear length, tear width, muscle atrophy, fatty degeneration, ROM, muscle strength, VAS score, and JOA score.

### Statistical analysis

JMP11 statistical software (SAS Institute, Cary, NC, USA) was used for statistical analyses. The Wilcoxon signed-rank test was used to compare between-group JOA scores pre- and postoperatively. Univariate logistic analysis was used to compare the relationship between the clinical parameters of the satisfactory and unsatisfactory groups and to analyze the relationship between the Goutallier stage in the rotator cuff muscles and sections in the JOA score. Multivariate logistic analysis using a stepwise technique was performed to evaluate the significant parameters affecting “satisfactory” or “unsatisfactory” outcomes in the JOA score, along with the odds ratio (OR) with 95% confidence intervals (CIs). Receiver operating characteristic (ROC) curve analysis was performed to obtain the cutoff value of the parameters affecting the clinical outcome. Data were expressed as mean values with standard deviations. *P* values < 0.05 were considered statistically significant.

## Results

Seventy-one patients were included in this study. The mean age at surgery was 63.4 ± 9.5 years. The mean symptom duration preoperatively was 10.1 ± 9.0 months.

**Fig. 1:**
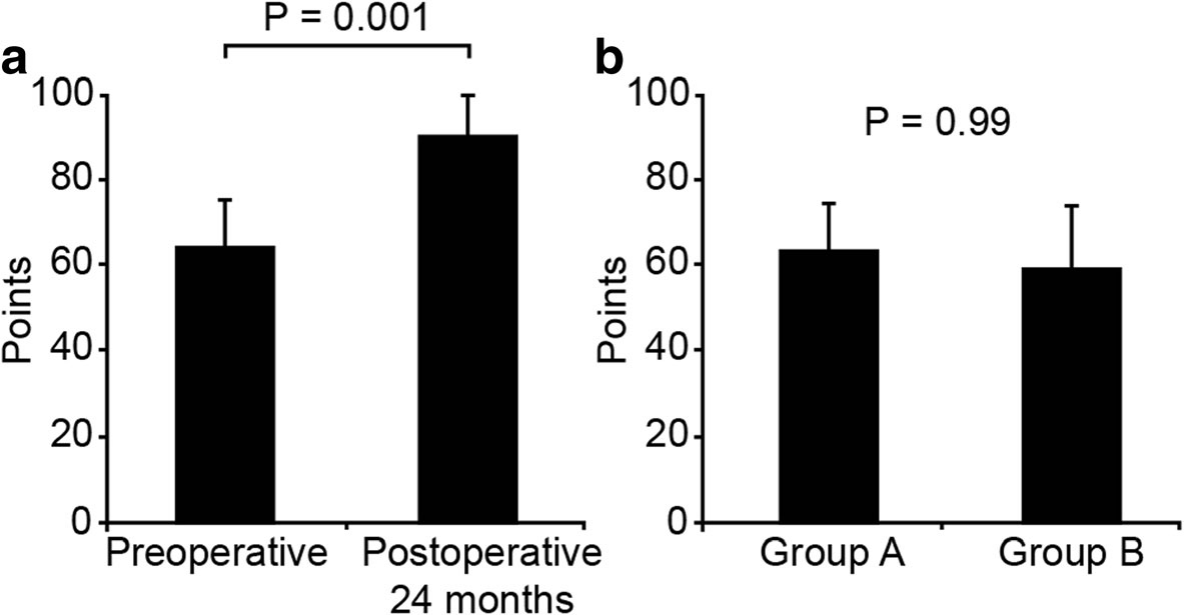
Mean JOA scores. **a** Mean preoperative and postoperative JOA scores of all patients. **b** Mean preoperative JOA scores of both the satisfactory (group A) and unsatisfactory (group B) group. Error bars represent standard deviation

The JOA score of all patients significantly improved from 63.7 ± 11.5 points preoperatively to 90.3 ± 9.6 points at 2 years, postoperatively (*P* < 0.05) (Fig. 1a).

The mean preoperative JOA scores were not significantly different between the two groups (Fig. 1b).

Re-tear postoperatively occurred in 10 patients at 3 months (9 patients) or 12 months (1 patient) postoperatively: 8 patients in group A and 2 in group B (*P* = 0.70).

### Univariate analysis to detect the factors affecting clinical outcomes

ROM (preoperative abduction, postoperative elevation, abduction, internal rotation external rotation), postoperative VAS (level 0 = no pain; level 10 = severe pain), Cofield classification, partial repair, and preoperative tear size on magnetic resonance images (width) (*P* < 0.05) (Table 1) were revealed as significant based on univariate analysis.

**Table 1:** Comparison of various variables between the satisfactory and unsatisfactory outcome groups

	Group A	Group B	*P* value
Age (years)	63.8	60.5	0.26
Sex (*n*)			
Male	35	9	
Female	24	3	0.31
Disease duration (weeks)	10.2	9.6	0.86
Trauma (*n*)	20	6	0.28
Contracture (*n*)	23	2	0.13
Diabetes mellitus (*n*)	6	1	0.85
Workmen’s accident (*n*)	5	1	0.95
Re-tear (*n*)	8	2	0.70
Preoperative ROM			
Elevation (°)	109	94	0.22
Abduction (°)	104	69	0.02^b^
Internal rotation (vertebrae)	4.3	4.5	0.90
External rotation (°)	41	37	0.47
Preoperative muscle strength			
Elevation	0.77	0.70	0.52
Abduction	0.72	0.51	0.10
Internal rotation	0.86	0.87	0.90
External rotation	0.69	0.58	0.39
Preoperative VAS	5.66	5.55	0.91
Postoperative (PO) ROM			
PO 2M elevation (°)	117	96	0.02^b^
Abduction (°)	104	85	0.09
Internal rotation (vertebrae)	2.1	1.0	0.08
External rotation (°)	29	16	0.03^b^
PO 3M elevation (°)	135	107	0.001^b^
Abduction (°)	126	92	0.003^b^
Internal rotation (vertebrae)	4.1	1.7	0.005^b^
External rotation (°)	36	23	0.024^b^
Postoperative (PO) muscle strength^a^			
PO 2M elevation	0.384	0.400	0.85
Abduction	0.377	0.455	0.44
Internal rotation	0.711	0.602	0.21
External rotation	0.514	0.533	0.86
PO 3M elevation	0.577	0.431	0.07
Abduction	0.595	0.481	0.21
Internal rotation	0.838	0.715	0.12
External rotation	0.765	0.523	0.004^b^
Postoperative (PO) VAS			
PO 2M VAS	3.01	5.75	0.001^b^
PO 3M VAS	2.46	4.71	0.012^b^
Radiographic findings			
Acromiohumeral interval (mm)			
Preoperative	9.57	9.1	0.64
Postoperative	12.16	12.42	0.75
Oizumi classification (*n*)			
Grade 0	32	3	
Grade 1	19	5	
Grade 2	6	2	
Grade 3	2	2	
Grade 4	0	0	0.10
Preoperative findings on MR images			
Cofield classification (*n*)			
Small	15	0	
Moderate	35	6	
Large	9	6	0.04^b^
Retraction (mm)	14. 31	17.77	0.18
Width (mm)	20.8	30.9	0.011^b^
Goutallier classification			
Supraspinatus (*n*)			
Stage 0	7	1	
Stage 1	27	4	
Stage 2	23	3	
Stage 3	2	3	
Stage 4	0	1	0.13
Infraspinatus (*n*)			
Stage 0	34	6	
Stage 1	19	5	
Stage 2	6	1	
Stage 3	0	0	
Stage 4	0	0	0.92
Subscapularis (*n*)			
Stage 0	39	5	
Stage 1	17	5	
Stage 2	3	2	
Stage 3	0	0	
Stage 4	0	0	0.20
Operative procedure (*n*)			
Suture bridge	54	11	
Single	4	2	0.77
Partial repair (*n*)	3	3	0.032^b^

Data were evaluated by logistic analysis

*ROM* range of motion, *VAS* visual analog scale

^a^Measured as percentage of unaffected side

^b^Statistically significant

### Multivariate analysis to detect factors affecting clinical outcomes and calculation of cutoff values

**Fig. 2:**
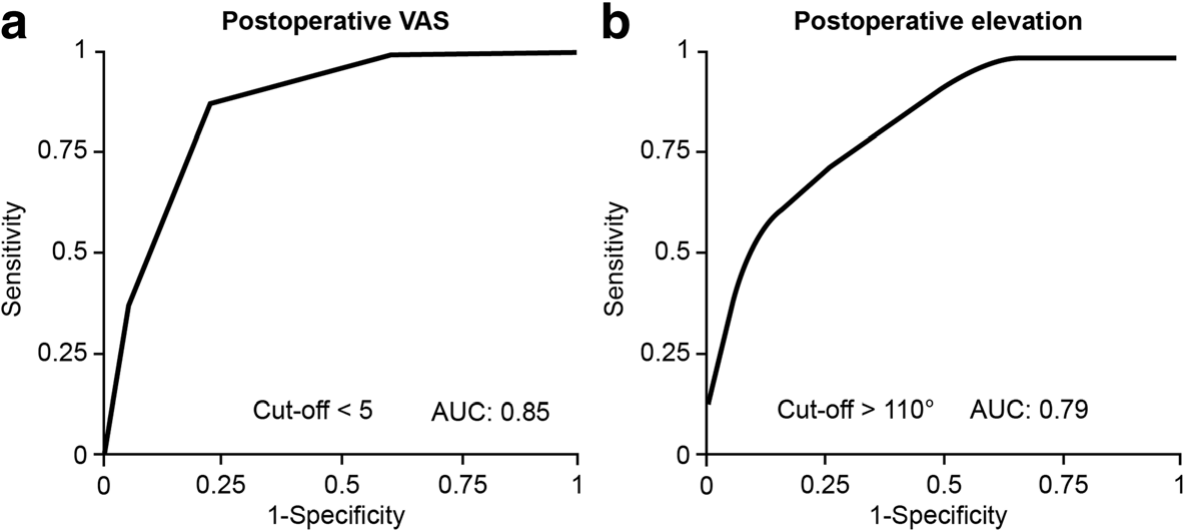
Receiver operating characteristic curve to determine the predictive cutoff value **a** for an unsatisfactory postoperative VAS score and **b** for postoperative elevation

Multivariate analysis and ROC curves showed that VAS < 5 points at 2 months (OR 0.46, 95% CI 0.21–0.79, *P* = 0.004) and elevation > 110° at 3 months, postoperatively (OR 1.08, 95% CI 1.03–1.18, *P* = 0.0005) were significant factors for obtaining a JOA score of 83 points or more at 2 years, postoperatively (Fig. 2a, b, Table 2).

**Table 2:** Variables associated with the outcome as verified by multivariate logistic analysis

	OR	95% CI	*P* value
Po2m VAS	0.46	0.21–0.79	0.004
Po3m elevation	1.08	1.03–1.18	0.0005

*VAS* visual analog scale, *Po* postoperative, *m* months

## Discussion

Postoperative outcomes of ARCR are generally favorable. However, the analysis of prognostic factors has included only preoperative factors, such as preoperative contracture, pain level, ROM, presence or absence of diabetes, thyroid disease [19], deep shoulder infection [20], and industrial accident [21–23]. However, although there are a few reports on postoperative factors, it has been reported that ROM, pain level, and re-tear at 3 months postoperatively affected the postoperative outcomes of rotator cuff repair (RCR) [4, 24]. In the present study, we analyzed how factors from preoperative to 3 months postoperative and re-tear affect JOA outcomes 2 years after ARCR. As a result, we found that active elevation at 3 months postoperatively (≥ 110°) and VAS at 2 months postoperatively (< 5 points) were significant prognostic factors. However, postoperative re-tear was not found to be a prognostic factor based on the analysis performed in the present study. Thus, owing to our analysis of the range, reported to up to 3 months postoperatively, including preoperative factors, it was suggested that postoperative factors are more important than preoperative factors.

Kim et al. [11] evaluated postoperative ROM at 6 weeks and 3, 6, and 12 months postoperatively using univariate and multivariate analyses and found that internal rotation at 3 months postoperatively affected the pain level at all time points (final observation at 12 months). Tonotsuka et al. [12] examined the association of ROM between 3 months and 2 years after surgery and reported that if an elevation of 120° or external rotation of 10° is not achieved at 3 months, then ROM is also decreased at 2 years postoperatively. In the present study, active elevation at 3 months postoperatively (≥ 110°) was identified as a significant factor in both univariate and multivariate analyses, which, in part, supports the report by Tonotsuka et al. [12].

Amako et al. [25] conducted a postoperative evaluation of rotator cuff tears in groups with and without preoperative pain and found a significant difference in the JOA scores and DASH scores up to 2 years postoperatively. They reported that persistent postoperative pain was a factor that reduces postoperative functional outcomes, patient level of satisfaction, and quality of life. They also reported that pre- and postoperative pains were closely related. In the present study, pre- and postoperative pains were included in our analyses. As a result, only postoperative pain was identified as a significant factor. Tonotsuka et al. [26] reported that it is important to control preoperative inflammatory pain to obtain better postoperative outcomes following rotator cuff repair. The results of the present study suggest that it is important to control postoperative pain. However, further examination is needed regarding the relationship between pre- and postoperative pain.

Postoperative re-tear is said to affect postoperative clinical outcomes. Chung et al. [27] reported that the acromiohumeral interval was a significant finding upon performing ARCR for extensive rotator cuff tears and performing evaluations based on the ASES score of 80 points. Furthermore, upon examining re-tear and clinical function in a recent meta-analysis, it was found that re-tear significantly reduced UCLA/ASES/Constant scores and that re-tear was a prognostic factor for clinical outcomes [13]. On the other hand, in the present study, re-tear did not affect postoperative outcomes. This should be further examined considering that the small sample size could have affected the results.

A significant difference in the number of partial repairs was observed based on the univariate analysis in the present study. If partial repair was considered a “structural failure” in re-tear cases, it was not a significant factor after multivariate analysis, although it could have fundamentally affected our results.

In the present study, multivariate analysis revealed that the pain level at 2 months postoperatively (VAS > 5) and the elevation of ROM at 3 months postoperatively (> 110°) affected the JOA score at 2 years postoperatively. This suggests that pain level and early ROM postoperatively are important factors to predict postoperative outcomes and simultaneously signifies that postoperative pain management, including attainment of early postoperative ROM, is important.

Shin et al. [28] reported that postoperative injection of steroids is useful in controlling pain for postoperative management. Cho et al. [29] administered postoperative local anesthetic transvenously or via the subacromial bursa and reported that it was useful in controlling pain. Nakagawa et al. [30] reported that postoperative cervical continuous epidural anesthesia for 2 weeks postoperatively significantly improved JOA score and pain evaluation at 3 months postoperatively. In our report, all patients received an anesthetic block only at the time of surgery, and postoperatively, they were only administered nonsteroidal anti-inflammatory drugs. Based on our results, we believe that postoperative pain management is important and better pain control is needed early postoperatively.

Shoulder-function rating systems for ARCR are used for the evaluation of clinical outcomes: UCLA score [31–33], Constant score [34], and ASES score [7, 31, 33, 35, 36]. Our recent study [18] shows that the cutoff value of 28 points in the UCLA score corresponded to 83 points in JOA score in patients with ARCR. Since there is growing evidence in the English literature about patients with ARCR evaluated by JOA score [32, 37–39], the present study focused on seeking prognostic factors affecting clinical outcomes after ARCR, exclusively using this score.

There are several limitations in the present study. First, this was a retrospective, small study. Second, evaluations solely comprised the JOA scores and did not include the patients’ perspectives; therefore, the data may have varied on the basis of the set standards. On the other hand, as a result of comprehensively analyzing preoperative factors to early postoperative factors reported to date, we conclude that pain level at 2 months postoperatively and elevation ROM at 3 months postoperatively have a greater effect than preoperative factors and that early postoperative intervention is important.

## Conclusions

To obtain a JOA score of ≥ 83 points at 24 months postoperatively, a postoperative VAS of < 5 points at 2 months and a postoperative elevation of ≥ 110° at 3 months should be achieved following ARCR.

## Abbreviations

ARCR: Arthroscopic rotator cuff repair
ASES: American Shoulder and Elbow Surgeons
CIs: Confidence intervals
JOA: Japanese Orthopedic Association
MRI: Magnetic resonance imaging
OR: Odds ratio
RCR: Rotator cuff repair
ROC: Receiver operating characteristic
ROM: Range of motion
UCLA: University of California Los Angeles
VAS: Visual analog scale
